# Temperature-driven biogeography of marine giant viruses infecting picoeukaryotes *Micromonas*

**DOI:** 10.1093/ismeco/ycaf137

**Published:** 2025-08-14

**Authors:** David Demory, Hisashi Endo, Anne-Claire Baudoux, Estelle Bigeard, Nigel Grimsley, Nathalie Simon, Hiroyuki Ogata, Joshua S Weitz

**Affiliations:** Sorbonne Université, CNRS, UMR 8176 Laboratoire de Biodiversité et Biotechnologies Microbiennes (LBBM), Observatoire Océanologique, Banyuls-sur-Mer, F-66650, France; Bioinformatics Center, Institute for Chemical Research, Kyoto University, Uji, 611-0011, Japan; Sorbonne Université, CNRS, UMR7144 Adaptation et Diversité en Milieu Marin (ECOMAP), Station Biologique de Roscoff, F-29680, France; Sorbonne Université, CNRS, UMR7144 Adaptation et Diversité en Milieu Marin (ECOMAP), Station Biologique de Roscoff, F-29680, France; Sorbonne Université, CNRS, UMR 8176 Laboratoire de Biodiversité et Biotechnologies Microbiennes (LBBM), Observatoire Océanologique, Banyuls-sur-Mer, F-66650, France; Sorbonne Université, CNRS, UMR7144 Adaptation et Diversité en Milieu Marin (ECOMAP), Station Biologique de Roscoff, F-29680, France; Bioinformatics Center, Institute for Chemical Research, Kyoto University, Uji, 611-0011, Japan; Department of Biology, University of Maryland, College Park, 20742, MD, United States; Department of Physics, University of Maryland, College Park, 20742, MD, United States; Institute of Health Computing, University of Maryland, North Bethesda, 20852, MD, United States

**Keywords:** marine viruses, phytoplankton, temperature, biogeography, Micromonas, TARA, ecological modeling

## Abstract

Climate shapes the biogeography of microbial and viral communities in the ocean. Among abiotic factors, temperature is one of the main drivers of microbial community distribution. However, we lack knowledge on how temperature shapes the life history traits, population dynamics, and the biogeography of marine viruses. This study integrates mathematical modeling with *in situ* observations to investigate the temperature-driven biogeography of marine viruses. We focused on prasinoviruses, a group of giant viruses that infect the picoeukaryote *Micromonas*, a widespread phytoplankton with thermotypes adapted from poles to tropics. Analyzing the Tara Oceans and Polar Circle databases, we found that temperature is the primary determinant of *Micromonas* virus (MicV) distribution in the surface ocean. Phylogenetic reconstruction of MicVs revealed that these viruses form several groups with cryophile or cryo-mesophile preferences. We applied a mechanistic model to describe temperature-driven population dynamics, allowing us to predict the global presence and absence of MicVs. The probability of lysis and the probability of infection emerged as reliable predictors of MicV distribution, indicating that temperature-driven cellular mechanisms significantly shape viral community structure and distribution in the global oceans.

## 1 Introduction

Viruses are drivers of microbial dynamics and regulate both ecosystems and biogeochemical cycles in the oceans [1, 2]. Among marine viruses, the phylum *Nucleocytoviricota* is a diverse viral group that includes families such as *Mimiviridae* and *Phycodnaviridae* [3, 4]. *Phycodnaviridae* are mostly lytic viruses characterized by large icosahedral capsids (up to 200 nm in diameter), with a lipid membrane, and contain large dsDNA genomes (up to 600 kb) [3]. They play crucial roles in the ocean by regulating the densities of their photosynthetic hosts (phytoplankton) and modulating ecosystem processes [5]. Through cell lysis, *Phycodnaviridae* alter the fate of organic carbon and can significantly increase or decrease carbon export to the deep ocean [6–9]. The infection of phytoplankton by phycodnaviruses begins with the attachment of the viral particle to the host cell, followed by the injection and replication of viral DNA and the assembly of viral particles using the host’s cellular machinery. The cycle ends with the lysis of the host cell and the release of new viral particles into the environment [10]. Each step of the lytic cycle can be characterized by viral life history traits (LHTs) that modulate the infection cycle, the host–virus population dynamics and the impact of viruses on the ecosystem [11, 12]. Given the variability of the ocean environment, the dynamics of phycodnaviruses and phytoplankton are strongly driven by environmental factors that modify LHTs through changes in host physiology, virus stability, and host–virus interactions [13, 14]. Among these factors, temperature stands out as one of the most critical determinants [15].

Temperature is a key driver of viral distribution in the ocean, with different viral communities occupying specific thermal niches [16–18]. At the population level, temperature shapes virus–host dynamics by modulating key viral life history traits, such as adsorption, latent period, burst size, and decay rates. Warmer temperatures typically accelerate viral replication but also increase viral decay rates [15, 19–22], ultimately influencing virus–host populations dynamics. Since virus and host metabolism are tightly linked, the viral response to temperature depends on the host’s thermal performance [15, 21, 22]. This interplay is best exemplified by prasinoviruses infecting the picoeukaryote *Micromonas*. Members of this genus are ubiquitous and abundant, especially in coastal and polar regions, where they contribute to global primary production and carbon cycling [23]. Their infecting prasinoviruses (MicV), large double-stranded DNA viruses, can significantly alter host population dynamics and drive biogeochemical cycles through viral lysis [24]. Near its optimal growth temperature, *Micromonas* supports efficient viral replication and rapid host lysis. At suboptimal temperatures, viral cycles are delayed, slowing host lysis. Above the optimal temperature, the viral lytic cycle and host cell lysis is significantly reduced [15]. It has been suggested that loss of viral infectivity at higher temperatures plays a key role in reducing viral lysis and altering virus–host dynamics [22].

Understanding how temperature influences viral LHTs and linking these effects to the distribution of viral populations is crucial in the context of global warming. Ocean warming may increase viral production by changing host physiology and their interactions with viruses [14, 15, 22, 25–27], but it can also lead to greater viral particle decay and their loss of infectivity [15, 22]. These opposing effects make it challenging to predict the impact of temperature on the distribution of viral populations. In a previous study, we proposed temperature-driven virus distribution patterns based on environmental niches where temperatures are favorable for viral growth [22]. Our interdisciplinary approach integrated experimental data with a temperature-driven model and allowed us to predict the distribution of viruses and the impact of climate change on the distribution of the virus community. We estimated the distribution of *Micromonas* virus will shift towards the poles due to the higher temperatures in the future tropics [22]. This virus–host pair represents an archetype model system to explore the impact of temperature on marine viruses *in situ* due to their cosmopolitan distribution and the variety of host thermotypes [28, 29]. Despite their ecological relevance, host–virus interactions involving prasinoviruses and *Micromonas* remain under-characterized.

In this paper, we integrate global metagenomes of *Tara* Oceans and Arctic expeditions with a mathematical model and explore the role of temperature in influencing MicV distribution, similar to the impact of temperature on host biogeography [28]. Phylogenetic analysis and viral distribution show that MicVs are grouped into cryo- and cryo-mesophile communities with distinct distribution patterns. Using our temperature-driven mathematical model, we simulate the distributions of MicV groups and find that the probability of infection and lysis are good indicators of the presence and absence of lytic prasinoviruses in the ocean. Our study emphasizes the value of combining mathematical modeling with experimental and environmental data to enhance our understanding of virus ecology in the ocean.

## 2 Materials and methods

### 2.1 Phylogeny and biogeography analyses

**Sample Collection:**  *In situ* samples were collected during the *Tara* Oceans expeditions [29]. A non-redundant gene catalog (Ocean Microbial Reference Gene Catalog version 2, OM-RGC.v2) was constructed from 370 metagenomic samples collected during these expeditions [30]. An abundance profile of *Nucleocytoviricota*, including MicV, was constructed based on the abundance matrix of the marker gene, family B DNA polymerase (PolB) [31]. An abundance matrix of eukaryotes was also generated using metabarcoding data of the V9 region of the 18S rRNA gene [32]. From these abundance matrices, samples from the pico-size fraction (0.22–1.6 $\mu $m or 0.22–3.0 $\mu $m) and the total size fraction (>0.8 $\mu $m) were used to generate abundance profiles of viruses and eukaryotes, respectively, as these fractions contained higher proportions of targets. Both abundance matrices were normalized to the total number of target communities.

**Recruitment of MicV Marker Genes:** Taxonomic classification of MicV PolB sequences derived from OM-RGC.v2 was conducted using EPA-ng [33], which is based on evolutionary placement algorithms (Supplementary Fig. S1). To generate the reference tree (backbone) of MicV, we used 22 full-length PolB sequences from MicV isolates and 64 long (>700 aa) environmental PolB sequences from OM-RGC.v2. A set of 41 short PolB sequences from MicV isolates with host information [21, 34] was also used to define viral groups in the reference tree to guide taxonomic affiliations. A maximum likelihood tree was constructed using the randomized accelerated maximum likelihood (RAxML) program (version 8.2.12) with the best-fitting amino acid substitution model (WGA) [35]. The environmental MicV PolB sequences were aligned with the reference sequences and then classified into 4 groups (A, B, C, and Pol) based on the maximum likelihood of their placement in the tree. Groups A, B, and Pol were further subdivided into A1, A2, B1, B2, Pol1, and Pol2. A total of 122 sequences were associated with reference MicVs. We manually checked the taxonomic assignments of these sequences by reconstructing the ML tree and removed 6 of the 122 sequences from downstream analyses, as they were placed far from the reference sequences. By comparing with the total *Nucleocytoviricota* polB abundance table [31], the relative abundances of MicV PolB sequences in *Nucleocytoviricota* were calculated for each latitudinal region.

**Recruitment of *Micromonas* Sequences:** The taxonomic assignment of 18S rRNA eukaryote gene sequences was performed using EPA-ng (Supplementary Fig. S2). The reference ML tree was built using 28 full-length 18S rRNA genes derived from *Micromonas* and an outgroup of Mamiellophyceae (Demory *et al.*, 2018) with RAxML (version 8.2.12) with the nucleotide substitution model (GTR). The environmental V9 18S rRNA gene sequences previously assigned to Mamiellophyceae were aligned with the reference sequences and then classified into *Micromonas* groups A (*M. commoda*), B (*M. bravo*), C (*M. pusilla*), and Pol (*M. polaris*). Groups A and B were further subdivided into A1, A2, B1, B2, and B3.

**Biomes Definition:** We defined five biomes according to the latitude ($lat$) and the sample temperature ($T$) in the *Tara* dataset: polar ($lat \geqslant 60^\circ $), temperate cold ($23^\circ \leqslant lat < 60^\circ $ and $T < 20^\circ $C), temperate warm ($23^\circ \leqslant lat < 60^\circ $ and $T \geqslant 20^\circ $C), tropical cold ($lat < 23^\circ $ and $T < 20^\circ $C), and tropical warm ($lat < 23^\circ $ and $T \geqslant 20^\circ $C).

**Geographical Viral Community Variation:** Community variation among samples was assessed using a Principal Coordinate Analysis (PCoA, function *pcoa* from the package *ape*) based on the Bray–Curtis dissimilarity matrix (function *vegdist* from the package *vegan*). Geographical community variation was verified using a permanova test (function *adonis2* from the package *vegan*) with 9999 permutations between polar and non-polar communities and between non-polar communities only. For each viral phylotype, the optimal temperature was defined as the temperature at which the maximum frequency was observed.

**Drivers of Viral Community Distribution:** Relationships between environmental variables and viral community distribution were analyzed using a linear regression model (function *lm* from the package *stats*). The environmental variables tested were nominal sample depth ($z$), mixed layer depth ($MLD$), temperature ($T$), salinity ($Sal$), phosphate ($P$), nitrite/nitrate ($N$), silicium ($Si$), and chlorophyll $a$ ($chla$). These variables were fitted to the first two dimension scores from the PCoA. The correlation between temperature and the first PCoA dimension was assessed by calculating the Pearson correlation coefficient (function *cor.test* from the package *stats*).

**Phylogenetic Tree Construction:** To construct a ML phylogenetic tree of *Micromonas* viruses, we used four sets of PolB sequences: 1) full-length PolB sequences of MicV isolates and their outgroups (Bathycoccus viruses and Ostreococcus viruses), 2) long (>700 aa) environmental PolBs from the OM-RGC.v2, 3) short PolB sequences from MicV isolates with host information [21, 34], and 4) a total of 122 environmental sequences recruited from the OM-RGC.v2 by EPA-ng analysis. These sequences were aligned with MAFFT (ver.7.487) [36] and the aligned sequences were trimmed with trimAl (ver.1.4.1) [37] using default settings. The tree was built with the best-fit substitution model LG+F+G4 and 1,000 iterations of ultrafast bootstrap approximation using IQ-TREE (ver.1.6.12) [38].

**Network Analyses:** We constructed a network based on co-occurrence patterns. We combined the abundance matrices of MicVs and their hosts. Then, infrequent phylotypes (observed less than 3 samples) and samples (having less than 3 phylotypes) were filtered out from the combined matrix. The resulting abundance matrix was normalized using centered log-ratio (clr) transformation after adding a small pseudocount. An interaction network was inferred using FlashWeave (ver.1.6.2) with sensitive mode and a threshold of $\alpha <0.01$. Among 430 significant pairwise associations, 420 positive pairs (97.7$\%$) were used for network visualization using the R package tidygraph (https://cran.r-project.org/web/packages/tidygraph).

### 2.2 Temperature-driven mathematical model

**Model Description:** We used a population model describing interactions between *Micromonas* and its prasinovirus [22]. Briefly, susceptible host cells ($S$) can be infected by infectious viral particles ($V_{i}$) and become infected ($I$). Infection can lead to lysis and the release of viral particles into the medium. Virus production includes infectious and non-infectious virus particles (i.e. defective viruses $V_{d}$). Virus particles can lose their infectivity and become defective. The model also accounts for basal losses of *Micromonas* and the decay of viruses due to first-order kinetic processes and higher-order losses (e.g. due to aggregation, binding to marine snow, and/or clearance by other populations). Operationally, we assume that higher-order viral loss terms are proportional to total virus densities, $V_{tot}=V_{i}+V_{d}$ as a form of nonlinear closure. The dynamics are described as follows:


(1) \begin{align*}& \begin{aligned} \frac{dS}{dt} &= \overset{\mathit{division}}{\overbrace{\mu S \left(1 - \frac{N}{K}\right)}} - \overset{\mathit{infection}}{\overbrace{\phi S V_{i}}} - \overset{\mathit{basal\ losses}}{\overbrace{\psi S}}, \\ \frac{dI}{dt} &= \overset{\mathit{infected}}{\overbrace{\phi S V_{i}}} - \overset{\mathit{viral\ lysis}}{\overbrace{\eta I}} - \overset{\mathit{basal\ losses}}{\overbrace{\psi I}}, \\ \frac{dV_{i}}{dt} &= \overset{\mathit{infectious\ viral\ production}}{\overbrace{(1 - \epsilon) \beta \eta I}} - \overset{\mathit{adsorption}}{\overbrace{\phi S V_{i}}} - \overset{\mathit{loss\ of\ infectivity}}{\overbrace{\sigma V_{i}}}\\ &\qquad- \overset{\mathit{higher-order\ viral\ losses}}{\overbrace{\omega V_{i} V_{\textrm{tot}}}}, \\ \frac{dV_{d}}{dt} &= \overset{\mathit{non-infectious\ viral\ production}}{\overbrace{\epsilon \beta \eta I}} + \overset{\mathit{loss\ of\ infectivity}}{\overbrace{\sigma V_{i}}} - \overset{\mathit{viral\ decay}}{\overbrace{\delta V_{d}}}\\&\qquad - \overset{\mathit{higher-order\ viral\ losses}}{\overbrace{\omega V_{d} V_{\textrm{tot}}}}. \end{aligned}\end{align*}

In this model, the interaction between viruses and their host is characterized by life history traits that are driven by temperature. The parameters of the model $\theta _{i}$ are mathematical functions driven by temperature such as $\theta _{i}(T) = f_{i}(T)$ where $f_{i}(T)$ are specific functional forms depending on the response of each trait to temperature. The model parameters are described in the Supplementary Table S1 and the functional forms driven by temperature can be found in [22].

**Estimation of Temperature-Driven LHTs:** We used parameters estimated by [22] for three virus–host pairs: Mic-A/MicV-A (RCC451/RCC4265), Mic-B/MicV-B (RCC829/RCC4265), and Mic-C/MicV-C (RCC834/RCC4229). Model fits and LHTs as a function of temperature for these three pairs can be found in [22]. Additionally, we fitted our model for one virus–host pair characterizing the polar region. We used experimental dynamics of *Micromonas polaris* strain TX-01 infected by virus MpoV45T from [21]. Similarly to [22], we used the MATLAB Differential Evolution algorithm [39] to estimate the best parameter set minimizing the error between model fits and experimental data for four temperatures: 0.5, 2.5, 3.5, and 7$^\circ $C. Host and virus fits are presented in Supplementary Figs S3 and S4, and LHTs as a function of temperature in Supplementary Fig. S5.

**Probability of Infection and Lysis:** Following [22], we computed the invasion fitness, the averaged number of newly infections due to the invasion of one virus particle in a virus-free host population, as follows:


(2) \begin{align*}& \mathcal{R}_{0} = \beta p_\epsilon p_\phi p_\eta\end{align*}

where $\beta $ is the burst size, $p_\epsilon $ is the proportion of infectious viral particles produced per lysed cell, $p_{\phi }$ is the probability of infection, and $p_{\eta }$ is the probability of lysis as follows:


(3) \begin{align*} & p_{\epsilon} = (1-\epsilon) \end{align*}

(4) \begin{align*} & p_{\phi} = \frac{\phi S^{*}}{\phi S^{*} + \sigma} \end{align*}

(5) \begin{align*} & p_{\eta} = \frac{\eta}{\eta + \psi} \end{align*}

where $\epsilon $ is the proportion of produced defective particles. $p_{\phi }$ is the probability that a virus infects a susceptible host cell before losing its infectivity, with $\phi $ being the adsorption rate, $\delta $ the viral decay rate, and $S^{*} = \left (1-\frac{\psi }{\mu }\right )K$ is the disease-free equilibrium calculated by considering no viruses in the environment such that higher-order loss terms are negligible, i.e. $\omega V_{tot}\approx 0$. Here, $\psi $ is the host basal mortality rate, $\mu $ the host net growth rate, and $K$ the host carrying capacity. $p_{\eta }$ is the probability that an infected cell lyses before dying due to other mortality processes, with $\eta $ being the lysis rate. More information on parameters can be found in Supplementary Tables S1 and S2. The relationship between model parameters and temperature involved in the estimation of $p_{\epsilon }$, $p_{\phi }$ and $p_{\eta }$ can be found in the Supplementary Text and in more details in [22].

**Receiver Operating Characteristic Analysis:** To estimate our model’s ability to predict the presence and absence of viruses in the *Tara* dataset, we performed a binary classification analysis using the R package *pROC*. First, for each viral group $i$ (A, B, C, and Pol), we classified the data as follows:


(6) \begin{align*}& p_{Tara,i}(x_{i})= \begin{cases} 1,& \textrm{if } x_{i}> 0\\ 0,& \textrm{if } x_{i} = 0 \end{cases}\end{align*}

where $x_{i}$ is the relative frequency of viral group $i$. Second, we computed the probability of lysis and infection, $p_\eta $ and $p_\phi $, given the temperature at each *Tara* station. We then computed the Receiver Operating Characteristic (ROC) curves using the function *roc*. We assessed the model’s ability to estimate presence and absence by computing the Sensitivity (or Recall) and Specificity rates as follows:


(7) \begin{align*} & \textrm{Sensitivity} = \frac{TP}{TP + FN} \end{align*}

(8) \begin{align*} & \textrm{Specificity} = \frac{TN}{TN + FP} \end{align*}

where $TP$ is the true positive rate, $TN$ is the true negative rate, $FP$ is the false positive rate, and $FN$ is the false negative rate. We also computed the area under the curve (AUC) and other statistics for our classification as follows:


(9) \begin{align*} & \textrm{Accuracy} = \frac{TP + TN}{TP + TN + FP + FN} \end{align*}

(10) \begin{align*} & \textrm{Precision} = \frac{TP}{TP + FP} \end{align*}

(11) \begin{align*} & \textrm{Sensitivity} = \frac{TP}{TP+FN} \end{align*}

(12) \begin{align*} & \text{F1 Score} = \frac{2 \times \textrm{Precision} \times \textrm{Sensitivity}}{\textrm{Precision} + \textrm{Sensitivity}} \end{align*}

Here, accuracy refers to how many positive and negative observations are correctly classified by the model. Precision refers to how many positive predictions are actually positive—also known as the positive predictive value. Sensitivity is the fraction of positively identified observations—also known as recall. The F1 score is the harmonic mean of precision and recall. AUC denotes the area under the ROC curve, which is used as a metric to evaluate the classification model We selected the optimal thresholds that maximize Sensitivity and Specificity based on the ROC curves for each viral group. The optimal temperature threshold was defined as the point that maximized the Youden Index (Sensitivity + Specificity – 1), providing the best discrimination between presence and absence.

**Global Distribution Model Estimation:** Following [22], we used global monthly averaged SST projections from the NOAA GFDL CM2.1 model (GFDL Data Portal: http://nomads.gfdl.noaa.gov/) run for the years 2010 to 2020. We defined the critical temperature of presence according to the probability of lysis threshold calculated from the ROC analysis for each MicV group. This temperature defined the maximum temperature of presence given the distribution of MicV groups in the *Tara* dataset. Additionally, we calculated the maximum temperature of growth where $\mathcal{R}_{0} = 1$. We mapped the model predictions of presence and absence using the MATLAB package $M_{Map}$ [40]. Model classification abilities were estimated using the ROC results and the classifications of true positive (TP), true negative (TN), false positive (FP), and false negative (FN) samples.

### 2.3 Statistical software

Computational analyses were performed using R (version 3.6.1, https://www.r-project.org/, [41]) and MATLAB (version R2023a, https://fr.mathworks.com, [42]).

## 3 Results

### 3.1 Temperature is a strong abiotic descriptor of the MicV distribution in the global ocean

**Figure 1:**
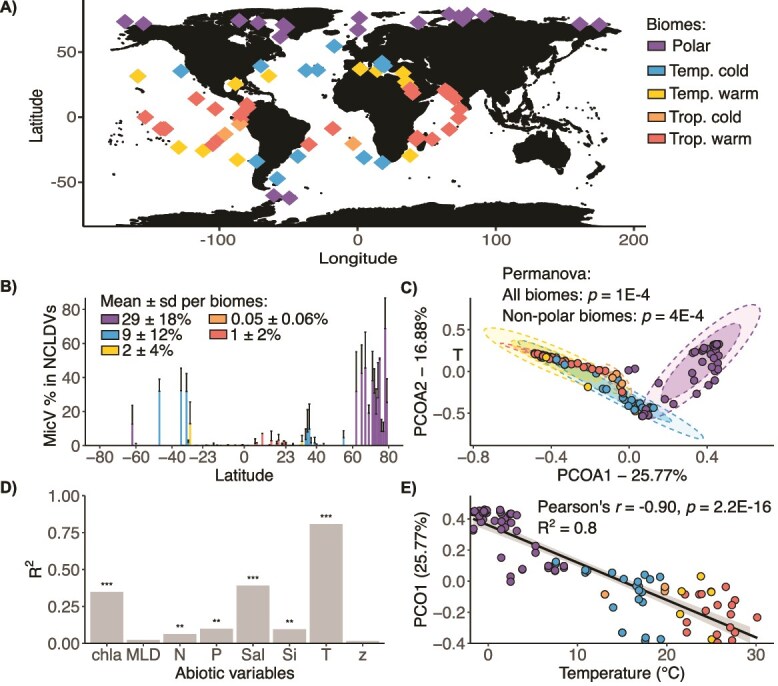
Environmental descriptors of MicV community distribution in global surface ocean. **A)** Location map of *Tara* Oceans expedition samples. **B)** Relative frequencies of MicV within the NCLDV (Nucleocytoplasmic Large DNA Viruses) community as function of latitude of the sample sites. **C)** Principal coordinate analysis (PCoA) with Bray–Curtis dissimilarity of the MicV communities. Ellipses represent the 75$\%$ and 95$\%$ CI of the centroids, respectively. **D)** Goodness of fit ($R^{2}$) of linear model of the first coordinate of PCoA vs. environmental variables: Chlorophyll a (chla), Mixed Layer Depth (MLD), Nitrates/Nitrites (N), Phosphates (P), Salinity (S), Silicates (Si), Temperature (T), and depth (z). **E)** Linear model of the first coordinate of PCoA vs. temperature. PCoA loadings can be found in Supplementary Table S3.

We explored the distribution of the MicV community in surface oceans using metagenomic data from the *Tara* Oceans and the *Tara* Polar Circle expeditions (Fig. 1a). After phylogenetic placement and manual curation, we defined 116 PolB sequences as *Micromonas* viruses (see Methods). MicV sequences were present globally and represented a large proportion of the *Nucleocytoviricota* community in temperate cold and polar biomes (Fig. 1b). MicV dominated the *Nucleocytoviricota* community in the Arctic Ocean with a proportion superior to 50$\%$ at many stations. In contrast, in warmer regions, MicV sequences represented only a small fraction of the *Nucleocytoviricota* community with proportions less than 5$\%$. Multivariate analysis revealed that MicV communities exhibit dissimilarities between biomes, especially between polar and other regions (Fig. 1c). Temperature, salinity, and chlorophyll *a* concentration were the main descriptors of the composition of the MicV community (Fig. 1d). However, temperature was by far the strongest descriptor, explaining 25$\%$ of the variance in the MicV composition (Fig. 1e and Supplementary Table S3). The MicV community displayed a gradient as a function of temperature, distinguishing between the 5 biomes: Polar, Temperate cold, Temperate warm, Tropcial cold, and Tropical warm.

### 3.2 MicV groups show either cryophile or cryo-mesophile preferences

**Figure 2:**
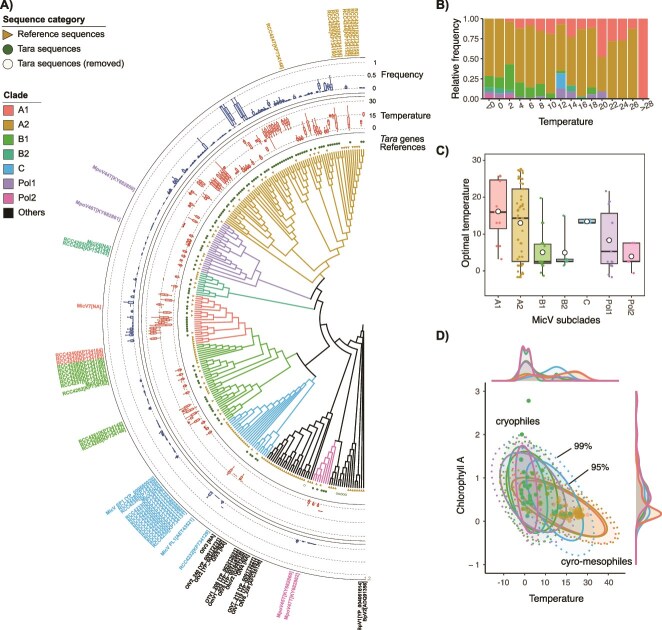
Relation between thermal distribution and MicV phylogeny. **A)** Phylogenetic affiliations of environmental MicVs. Reference and environmental PolB sequences are shown in triangle and circle marks, respectively, at the first layer. Environmental sequences placed on other virus lineages were removed from the analysis and shown in open circles in this plot. Box plots in the second and third layers represent the temperature range where each phylotype exists and the relative frequency, respectively. The outside layer indicates the phylogenetic positions of MicV strains having clade information. **B)** Relative frequency of MicV subclades as function of temperature. **C)** Optimal temperature of MicV subclades distributions. Boxplot edges are the 25$\%$ and 75$\%$ quantiles, horizontal black lines are the means, white circle are the medians, and the vertical black lines are the 5$\%$ and 95$\%$ quantiles. **D)** Distribution of MicV subclades across environmental gradients of chlorophyll A concentration and temperature at isolation sites. Ellipses represent the 95$\%$ and 99$\%$ CI of the centroids for each subclades. Data points in **B)** and **D) represent individual values.**

We reconstructed a phylogenetic tree of MicVs using reference and environmental PolB sequences to find relationships between MicV lineages and their thermal environment (Fig. 2a). Reference MicV sequences with known host species [34] were used to classify various MicV lineages (Supplementary Fig. S1). Viruses infecting *Micromonas pusilla* were found to form a monophyletic group C. In contrast, MicVs that infect other *Micromonas* species did not form monophyletic groups. Viruses of *Micromonas commoda* were placed in two groups (A1 and A2), those of *Micromonas bravo* were in two groups (B1 and B2), and those of *Micromonas polaris* were in two groups (Pol1 and Pol2). Although each MicV clade mainly infects the corresponding host clade, some MicV clones can infect a wide range of host clades [21, 34]. For example, the MicV Pol clone MpoV-44T can not only infect *M. Polaris*, but also species *M. commoda* and *M. pusilla* [21].

Group A largely dominates the MicV composition with relative frequency within the MicV community over 50$\%$ in all temperatures while other groups are present only at temperatures below 20$^\circ $C (Fig. 2b). Group A2 dominates the composition up to 26$^\circ $C, and group A1 is the only group present at temperatures above 28$^\circ $C. Groups B and Pol represent together between 25$\%$ and 40$\%$ of the MicV community at low temperatures, and group C is the least frequent MicV group. MicV groups display significant variability in their optimal environmental temperature (Fig. 2c). Group A1 has an optimal temperature ($^\circ $C) at 17.07 $\pm $ 6.28 (n = 13) with a lower variance compared to A2 (12.91 $\pm $ 10.01, n = 55). Group C also has an optimal warm temperature at 13.42 $\pm 0.52$ (n = 5) with a narrow thermal range. Groups B1 and B2 have low optimal temperatures with 4.79 $\pm $ 4.77 (n = 18) and 4.97 $\pm $ 5.65 (n = 5), respectively. Finally, the Pol1 and Pol2 groups also have low optimal temperatures with 7.66 $\pm $ 8.65 (n = 15) for Pol1 compared to 3.92 $\pm $ 3.54 (n 5) for Pol2, though Pol1 has a wider distribution than Pol2. MicV groups A have a cryo-mesophile profile and are present in lower chlorophyll *a* environments, while groups B and Pol are cryophiles and present in environments with high and low chlorophyll *a*. Group C has a more mesophilic profile, but is present in only a few stations (Fig. 2d).

### 3.3 MicV and their *Micromonas* hosts have similar environmental optimal temperatures

**Figure 3:**
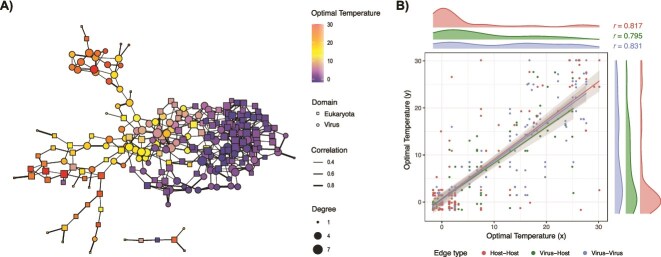
Inference of a geographical association-derived virus–host interaction network. **A)** Network is color-coded by the optimal temperatures of viruses (circle) and hosts (square). Edge width and node size indicate correlation coefficient and degree (i.e. the number of connections), respectively. **B)** Relationship between the optimal temperatures of host–host ($n=190$), virus–host ($n=103$), and virus–virus ($n=127$) pairs having positive associations in the network. All edge types (node category combinations) represent significant positive correlations at $p < 0.01$.

To further assess the relationship between temperature and the distribution patterns of viruses and hosts at the phylotype level, we conducted a co-occurrence network analysis (see Methods for details). Following convention in biogeographic studies of giant viruses and their eukaryotic hosts [31], and in contrast to the potential misinterpretation of associations in time series dynamics [43], we interpret positive associations as a potential indicator of interactions [44]. Hence, we construct an interaction network with 101 viruses and 140 hosts. A total of 430 pairs (1.5$\%$ of possible pairs) of phylotypes were found to have significant associations, with 420 pairs (97.7$\%$) being positive associations (Fig. 3a), including 103 virus–host interactions, 127 virus–virus interactions, and 190 host–host interactions. The viruses and hosts assigned to the same clade were arranged adjacent to each other in their respective temperature ranges (Fig. S6). For all network edges (that is, positive associations), significant positive correlations were found between the optimal temperatures of the connecting phylotypes (Fig. 3b). Host–host associations were concentrated in low-temperature regions, while virus–host and virus–virus pairs were more uniformly distributed throughout the temperature range.

### 3.4 A temperature-driven model shows good predictive ability for presence and absence of MicV groups

**Figure 4:**
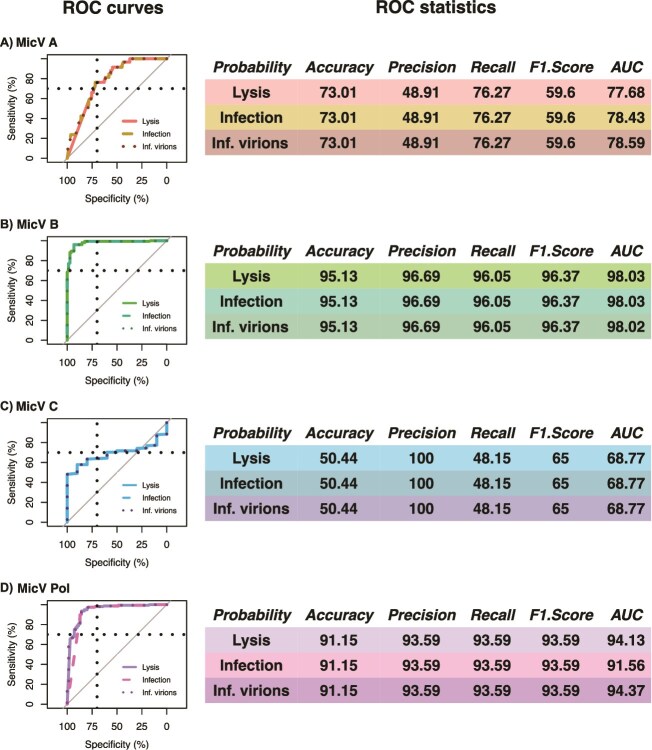
ROC analysis using the probability of lysis and of infection and the proportion of infectious produced virions estimated from mathematical modeling for MicV groups **A)** A, **B)** B, **C)** C, and **D)** Pol. Left panels are the ROC curves for probability of lysis (colored solid line), probability of infection (colored medium-dashed line) and proportion of infectious produced virions (colored small-dashed line). Vertical and horizontal dashed black lines are the 70$\%$ threshold of Specificity and Sensitivity. Gray 1:1 curve represents models that cannot distinguish between presence and absence in data. Right panels are ROC statistics with Accuracy (how many positive and negative observations are correctly classified), Precision (how many positive estimations are actually positive), Recall or Sensitivity (how many positive observations are classified correctly), F1 score (harmonic mean of precision and recall), and AUC (area under the curve)—see Methods for more details.

A mathematical model of temperature impacts on virus-phytoplankton dynamics [22] was used to compare theoretical predictions with the environmental presence of MicV groups in the *Tara* dataset (See methods for more details). We used the probability of lysis, the probability of infection, and the proportion of infectious virions produced as indicators of MicV group presence or absence, and estimated threshold values for each indicator using Receiver Operating Characteristic (ROC) analyses to discriminate between presence and absence of MicV groups (Fig. 4—see Methods for more details). The probability of lysis and infection and the proportion of infectious virions were good indicators of the presence and absence of MicV group A (accuracy > 0.7, good prediction) and group B and Pol (accuracy > 0.9, excellent prediction) but failed for group C (accuracy = 0.5). It is likely that the model failed to predict the distribution of MicV group C due to the small number of sequences in the *Tara* data (only found in 10 stations of 226), but also because viruses from this group have longer latent periods (up to 30 hours), which could reduce the probability of lysis.

### 3.5 Global model prediction reveals the importance of lysis as a driver of the MicV distribution

**Figure 5:**
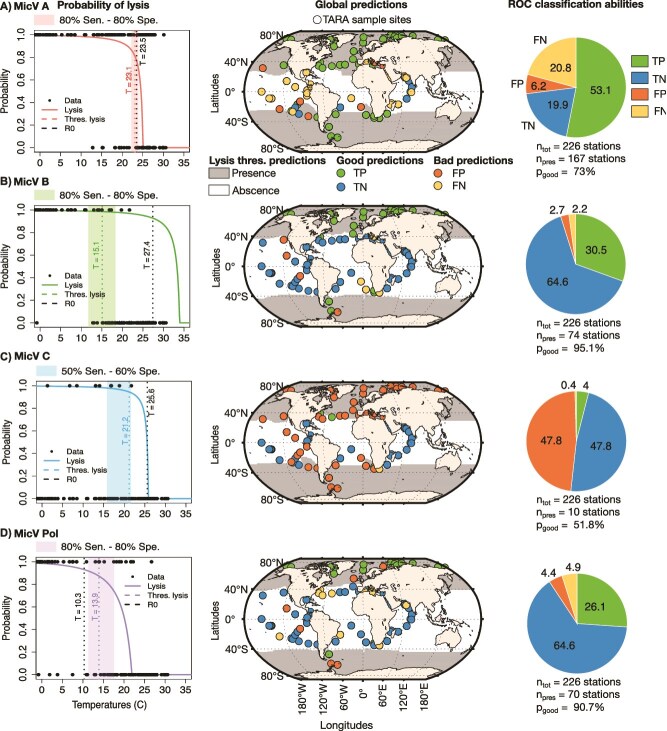
Model estimation of MicV global distribution based on the probability of lysis for MicV groups **A)** Clade A, **B)** Clade B, **C)** Clade C, and **D)** Clade Pol. Left panels are the probability of lysis (solid line) as function of temperature. Black dots are *Tara* presence and absence data. We considered presence when the relative frequency of each group is positive and absence when it is null. Vertical dashed black and colored lines are the temperature threshold estimated using $\mathcal{R}_{0}$ and probability of lysis. Shaded areas represent the threshold temperature for the optimum sensitivity (Sen) and specificity (Spe) with 70$\%$ Sen—70$\%$ Spe for group A, 80$\%$ Sen—80$\%$ Spe for groups B and Pol, and 50$\%$ Sen—60$\%$ Spe for group C. Middle panels are the global map estimation of presence and absence of MicV groups. Gray areas are the presence regions estimated with the best threshold for the probability of lysis. Dots are *Tara* samples with colors based on their classification: True Positive (TP–green), True Negative (TN–blue), False Positive (FP–orange), and False Negative (FN–yellow). Right panels are the classification percentage for TP (green), TN (blue), FP (orange), and FN (yellow). $n_{tot}$ is the total number of *Tara* samples, $n_{pres}$ is the number of *Tara* samples that have presence observations, and $p_{good}$ is the percentage of good estimation of presence and absence (TP+TN) by our model.

Given the similar predictions of our 3 indicators, we used the probability of lysis as predictor of the presence or absence of MicV in the global surface ocean (Fig. 5). We estimated maximum temperature thresholds from the ROC analysis and compared them to the maximum temperature of invasion fitness, when $\mathcal{R}_{0}> 1$ for the four MicV groups A, B, C, and Pol (Fig. 5 left panels). The threshold temperature for group A is similar to the maximum temperature of invasion (23.1$^\circ $C and 23.5$^\circ $C, respectively). For MicV group B, we find significant differences between the two temperatures, with 15.1$^\circ $C and 27.4$^\circ $C for the threshold and invasion temperatures, respectively. The differences are less significant for group C and Pol, with 21.2$^\circ $C and 25.6$^\circ $C for the former and 10.3$^\circ $C and 13.9$^\circ $C for the latter. We map the presence and absence regions according to the temperature threshold for the four groups and quantify the classification ability of our model by comparing the *Tara* data with our model estimates (Fig. 5 middle and right panels). Our model estimates the presence of MicV group A from the polar region to more than 40$^\circ $ latitude with an accuracy of 73$\%$ for true positive and true negative samples. Specifically, 53$\%$ of the samples are classified as true positive and 19.9$\%$ as true negative, while 6.2$\%$ and 20.8$\%$ are classified as false positive and false negative, respectively. The false negative samples are distributed mainly in the warmer tropical regions. The distribution of group B ranges from the polar region to a maximum of 40$^\circ $ latitude, with 95.1$\%$ of the samples having a good classification. True positive samples represent 30.5$\%$ and true negative 64.6$\%$, while only 2.7$\%$ and 2.2$\%$ are classified as false positive and false negative. Similarly to group A, group C has a wide distribution from the polar region to more than 40$^\circ $ latitude, but the precision of our model is poor. True positives represent only 4$\%$ of the samples and true negative 47.8$\%$, while 47.8$\%$ of the samples are classified as false positive and 0.4$\%$ as false negative. False positive samples are distributed mainly in cold environments beyond 40$^\circ $ latitude. The Pol group has an accuracy of 90.7$\%$ and is distributed up to 40$^\circ $ latitude. True positive samples represent 26.1$\%$ and 64.6$\%$ are classified as true negative. False positive and negative account for only 4.4$\%$ and 4.9$\%$, respectively.

## 4 Discussion

In this study, we explored the temperature-driven biogeographic pattern of viruses infecting the picoeukaryote *Micromonas* by integrating environmental metagenomics and mathematical modeling. We showed that temperature is the main driver of the distribution of the MicV community in the global ocean; specifically, we identified cryophile and cryo-mesophile phylogenetic viral groups, following the thermal niches of their hosts. Using a temperature-driven dynamical model of host–virus interactions, we estimated the viral abilities to infect and lyse their host for each viral group as a function of environmental temperatures and predicted the presence and absence of MicV groups. Our modeling predictions matched the presence and absence patterns for most viral groups in the *Tara* dataset.

Our analysis shows that model fits of the distribution of the MicV community are mainly driven by temperature. This result is in line with previous studies showing that temperature is one of the main drivers of dsDNA giant virus [18, 31], dsDNA phage [32], jumbo phage [45] and RNA [46], and DNA virome biogeography [16, 17]. Interestingly, we found a strong difference between the composition of the polar and non-polar MicV community and that MicV dominates the *Nucleocytoviricota* sequences in the Arctic ocean. Meng and colleagues [18] also reported that the cold temperatures of the Arctic Ocean act as an ecological barrier that separates the polar and non-polar giant virus communities. This ecological barrier might drive viral speciation and contribute to the high viral diversity observed in the Arctic Ocean [18, 32]. Temperature not only separates polar and non-polar MicV communities but also describes a gradient in their community composition from temperate cold to tropical warm water, explaining up to 23$\%$ of the variance in our dataset. Other abiotic factors significantly influenced the composition of MicV, such as chlorophyll *a* and salinity, but had a lower impact. Chlorophyll *a* is often used as a rough proxy for phytoplankton biomass [47, 48] and suggests that host abundances are also driving the biogeography of MicV. Host abundances are highly variable in coastal areas where chlorophyta (especially *Micromonas*) often dominate the phytoplankton community [49, 50], suggesting that they might play an important role in the dynamics of MicV [34].

Despite the strong influence of temperature on the distribution of MicVs, their phylogenetic groups cannot be defined as true thermotypes, unlike their host *Micromonas*, for which thermal adaptation aligns more clearly with phylogeny [28]. MicVs that infect similar hosts *Micromonas* are found in phylogenetically distinct groups, indicating that closely related viruses can exhibit different thermal responses, while phylogenetically distant viruses may share similar host associations. For example, subgroup A1 is genetically closer to subgroups B1 and C, while subgroup A2 is more closely related to Pol1. This suggests that MicV thermal traits are shaped more by host–virus interactions than by viral evolutionary lineage alone. This result does not necessarily mean that virus thermotypes do not exist, but that the taxonomic level of our analysis might be insufficient or that the ability of some viral groups to infect different host thermotypes is certainly blurring the signal [34]. In addition, viral groups B and Pol can be characterized as cryophilic, as the mean of their temperature distribution across samples is below 10$^\circ $ C, while group A has a wide thermal distribution extending to temperatures higher than 25$^\circ $C. Within each group, we also identify various thermal optima, suggesting that genetically closely related viruses can have different responses to temperature, depending on how they interact with their host [14]. Within group A, subgroup A2 has a wide temperature distribution, and dominates the MicV community from 0$^\circ $ to 26$^\circ $C. Subgroup A1 is not present lower than 10$^\circ $C, suggesting that it is more adapted to warm environments. The fact that model predictions include high false negative rates for the MicV group A distribution prediction in the tropics might suggest that MicV group A has a higher potential for adaptation to high temperature and can invade warmer environments. Our findings highlight the complexity of viral thermal responses and suggest that further explorations of the mechanisms driving these patterns are necessary.

Temperature affects viral fitness and distribution through various biological processes, with different impacts at low and high temperatures. At low temperatures, viruses degrade more slowly, suggesting that viral fitness is limited by the host cell’s metabolism and lower growth rate [15, 22]. Factors like the variability of *Micromonas* species in growing at low temperatures [28] and abiotic factors such as salinity affect how well the host can support viral replication [51]. Specific patterns of infection among virus groups [34] and the impact of coinfection (multiple viruses infecting the same cell) [52] could also explain why certain virus groups, such as MicV group A2, dominate, while others are less prevalent. MicV groups A and B can infect the same *Micromonas* species (*commoda* and *bravo*), leading to competition at the extracellular and intracellular stages. Outside the host cell, viral particles compete to infect susceptible hosts; within the host cell, coinfection can result in direct competition between viruses of different groups. These two levels of competition enhance the ecological interactions between MicV groups A and B and may contribute to the observed differences between their realized and fundamental thermal niches. If subgroup A2 exhibits greater competitive fitness than other viral groups, it could outcompete them and dominate under those temperature conditions. In contrast, at higher temperatures, rates of viral particle degradation and loss of infectivity are high, suggesting that viral fitness is mostly driven by degradation processes [15, 22]. The mechanisms driving viral loss of infectivity in response to temperature are not yet well characterized. Temperature can increase the virus polymerase error rate [53], resulting in the production of defective viral particles [22, 54]. These defective particles can compete with wild-type virus, potentially reducing their fitness and reducing the ability of viruses to extend their thermal niches in warmer environments. The distribution of MicVs suggests that these viruses may adapt and evolve more easily to colder rather than warmer environments as previously proposed [18, 22] - though understanding virus evolvability *in situ* remains an open challenge.

Our integrative approach allows for good to excellent predictions of the majority of MicV groups. The probability of lysis and infection and the proportion of infectious produced virions emerge as strong predictors of virus distribution. Identified MicVs are lytic viruses [24], and therefore, the presence of viral genetic signatures in metagenomes increases with the virus ability to infect and lyse their host. The difference between the temperature threshold for viral presence (estimated by ROC analysis and referred here as $T_{R}$) and the maximum temperature at which viral growth is still possible ($\mathcal{R}_{0} = 1$, referred as $T_{F}$) highlights a discrepancy between the realized and the fundamental thermal niches of these viruses. In ecology, the fundamental niche refers to the full range of environmental conditions under which an organism can survive and reproduce, in the absence of limiting biotic interactions such as competition. The realized niche, by contrast, reflects the conditions under which the organism is actually found—often a narrower subset constrained by ecological interactions [55]. For example, MicV group A’s $T_{R}$ is close to its $T_{F}$, suggesting that it occupies most of its thermal potential. In contrast, MicV group B’s $T_{R}$ is 12$^{\circ }$C lower than its $T_{F}$, indicating a considerable restriction in its actual distribution. This mismatch may be driven by ecological constraints such as competition, especially since both MicV groups A and B infect the same hosts (*Micromonas commoda* and *bravo*), leading to potential host competition [34]. This result suggests that MicV group A might have higher competitive fitness than MicV group B and can extend its realized niche to the maximum theoretical limits of its fundamental niche. In contrast, MicV group B is limited to a lower maximum temperature, where the competitive pressure from MicV group A (especially subgroup A1, which is not present at low temperatures) may be reduced. MicV group A might have better competitive fitness at high temperatures by reducing viral loss of infectivity, minimizing the production of defective particles through cell lysis [22], or better managing co-infection. Indeed, a recent study proposed that interspecific co-infection drives virus–virus competition in *Micromonas* viruses and that shifts in temperature alter competition outcomes [52]. Our study highlights the importance of investigating the impact of temperature on interspecific competition mechanisms.

The temperature-driven mathematical model is able to predict the presence and absence of viruses across the global oceans, albeit with caveats. First, on the experimental side, our model is parameterized on only four virus–host pairs, and we lack experimental studies investigating the intraspecific temperature response within each thermal group. Therefore, we may be missing potential intraspecific variability that could be reflected by some false positive and negative rates in our predictions. To extend our approach to other phytoplankton and virus genera, we also need experimental studies investigating the impact of temperature on virus–host dynamics at different taxonomic levels. Secondly, on the observational side, the *Tara* dataset is biased toward the Northern Hemisphere, especially for the polar region, while the Southern Hemisphere is underrepresented [30]. Increasing sampling in the Antarctic region will help to achieve better estimations and validations of virus biogeography. Furthermore, the low presence of MicV group C suggests that our reference sequences may not be large enough to recruit target species from the metagenomes. This highlights the need to expand the full length of PolB sequences, other marker genes, or genomes having host information for better distinguishing intra-genus and intra-species virus groups.

In conclusion, our study represents an attempt to link virus temperature responses to virus biogeography using an integrative mechanistic approach rather than a solely correlative one [56]. We highlight the importance of temperature in shaping virus ecology and biogeography, but also emphasize that interdisciplinary studies are needed to gain a better understanding of the impact of other processes on virus ecology.

## Supplementary Material

SUPPLEMENTARY_ISMECOMMUN-D-25-00040R1_v2_ycaf137

## Data Availability

The codes and datasets generated during the current study are available in the ZENODO repository: 10.5281/zenodo.14411352
