# Supplementary material for: Temperature-driven biogeography of marine giant viruses infecting picoeukaryotes Micromonas
Source: ISME Commun. 2025 Aug 14;5(1):ycaf137. doi: 10.1093/ismeco/ycaf137 (PMC12448749; doi:10.1093/ismeco/ycaf137)
Supplement: SUPPLEMENTARY_ISMECOMMUN-D-25-00040R1_v2_ycaf137 [file supplementary_ismecommun-d-25-00040r1_v2_ycaf137.pdf]

# Supplemental Information

## Supplementary Text: Model functions in response to temperature

We describe the temperature-dependent functions used to estimate $p_\epsilon$, $p_\phi$, and $p_\eta$ in the following equations. More information on the full model can be found in [22].

### Proportion of infectious viral particles produced per lysed cell ($p_\epsilon$):

$$p_\epsilon(T_K) = (1 - \epsilon(T_K)) \tag{S1}$$

where $T_K$ is the temperature in Kelvin, and $\epsilon(T_K)$ is the proportion of defective particles produced, which varies as a function of temperature as follows:

$$\epsilon(T_K) = \frac{1}{1 + exp\left(\frac{-d2}{T_K}\left(1 - \frac{T_\epsilon}{T_K}\right)\right)^{\epsilon_r}} \tag{S2}$$

where $d_2/T_K$ is the rate of protein degradation, $T_\epsilon$ is the temperature at which 50% of the produced viruses are non-infectious, and $\epsilon_r$ is a scaling constant.

### Probability of infection ($p_\phi$):

$$p_\phi(T_K) = \frac{\phi(T_K)S^*(T_K)}{\phi(T_K)S^*(T_K) + \sigma(T_K)} \tag{S3}$$

where $\phi(T_K)$ and $\sigma(T_K)$ are the adsorption rate and the loss of infectivity, respectively, both varying with temperature:

$$\phi(T_K) = \frac{\phi_K}{\phi_K + exp\left(-\phi_r(T_K - T_\phi)\right)} \tag{S4}$$

where $T_\phi$ is the half-saturation temperature, $\phi_r$ is the exponential rate, and $\phi_K$ is the saturating adsorption value.

$$\sigma(T_K) = \sigma_1 \, exp\left(\frac{-d2}{T_K}\right) \tag{S5}$$

where $d_2/T_K$ is the rate of protein degradation and $\sigma_1$ is a scaling coefficient.

The disease-free equilibrium function in response to temperature is described as follows:

$$S^*(T_K) = \left(1 - \frac{\psi(T_K)}{\mu(T_K)}\right)K \tag{S6}$$

where $\mu(T_K)$ and $\psi(T_K)$ are the gross phytoplankton growth and non-lysis mortality functions of temperature, respectively, and $K$ is the carrying capacity, assumed to be constant with respect to temperature:

$$\mu(T_K) = A_1\, exp\left(\frac{-E1}{T_K}\right) \tag{S7}$$

$$\psi(T_K) = A_2\, exp\left(\frac{-E2}{T_K}\right) \tag{S8}$$

where $A_1$, $A_2$, $E_1$, and $E_2$ are constants.

**Probability of lysis ($p_\eta$):**

$$p_\eta(T_K) = \frac{\eta(T_K)}{\eta(T_K) + \psi(T_K)} \tag{S9}$$

where $\eta(T_K)$ is the lysis rate, which varies as a function of temperature:

$$\eta(T_K) = s_1\, exp\left(\frac{-s_2}{T_K}\right) - d_1\, exp\left(\frac{-d_2}{T_K}\right) \tag{S10}$$

where $s_1$, $s_2$, $d_1$, and $d_2$ are constants. The first exponential term represents viral protein synthesis, and the second represents viral protein degradation.

## Supplementary Figures

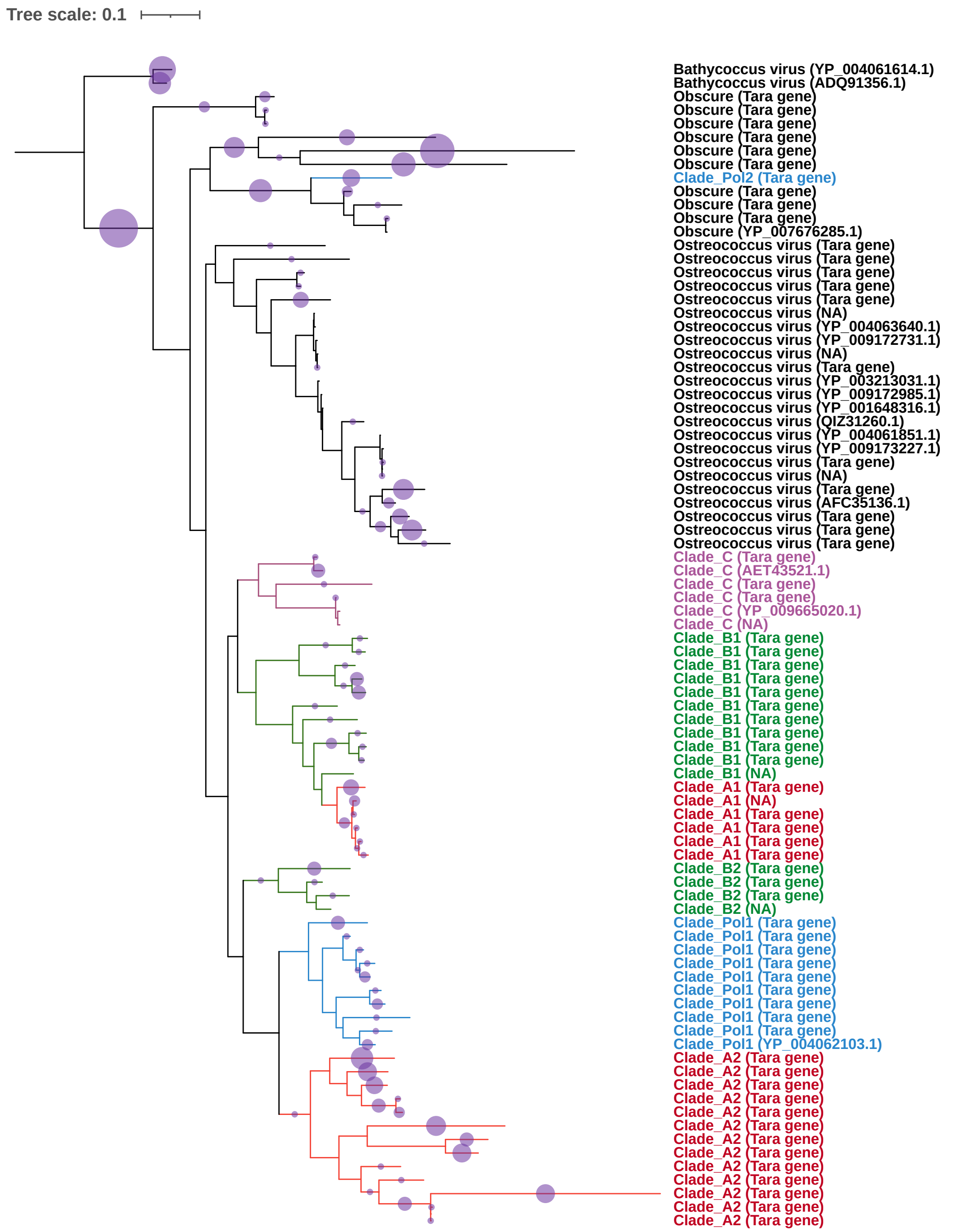


**Figure S1.** MpV phylogenetic tree of full-length or long (>700 aa) PolB sequences of Prasinoviruses used as the EPA-ng analysis. Taxonomic affiliations were confirmed by the previously published short sequences in [34, 21].

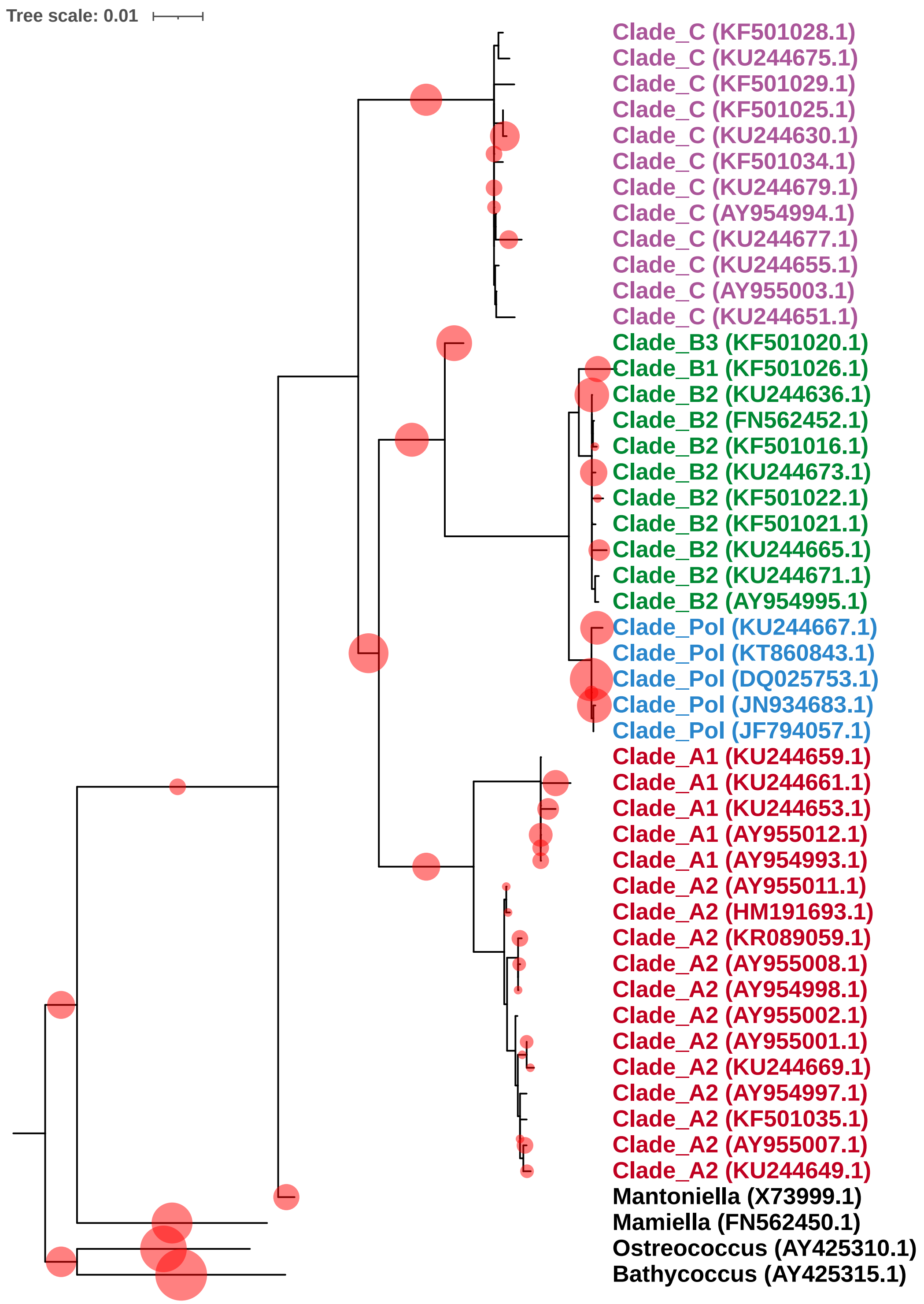


**Figure S2.** Micromonas phylogenetic tree of full length 18S rRNA gene sequences of Micromonas used as the EPA-ng analysis. Taxonomic affiliations were confirmed by the previously published short sequences in [28].

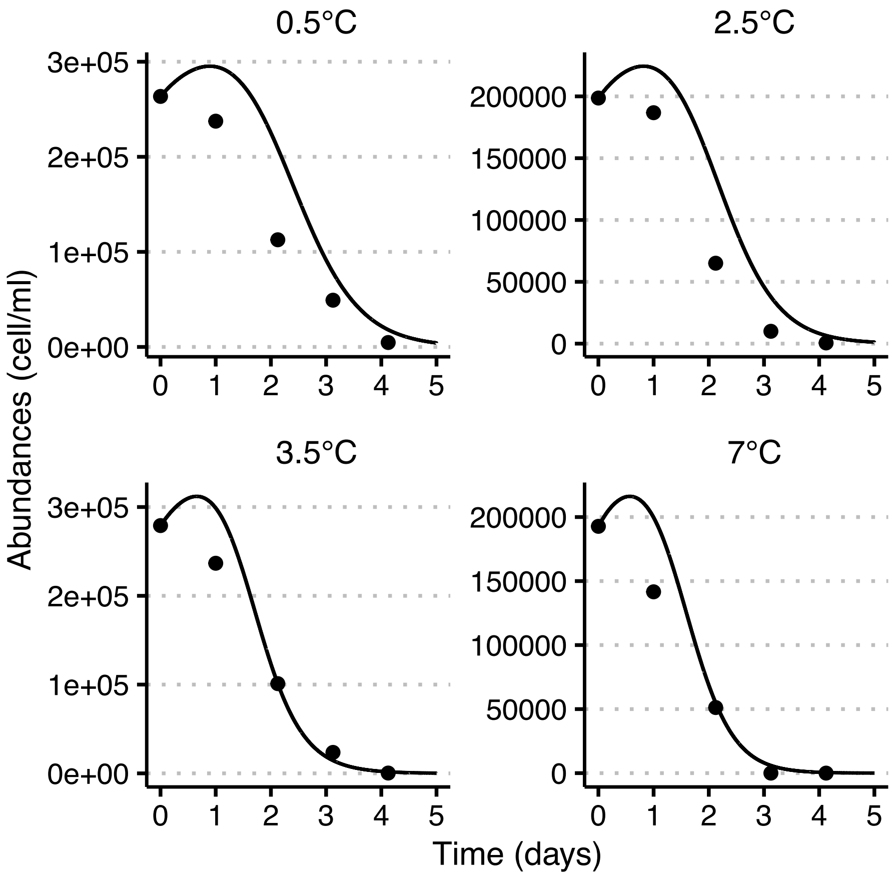


**Figure S3.** Model-data fitting for the polar virus-host pair. Host dynamics at 0.5, 2.5, 3.5 and 7°C. Solid black lines are the model fits and black dots the data from [21].

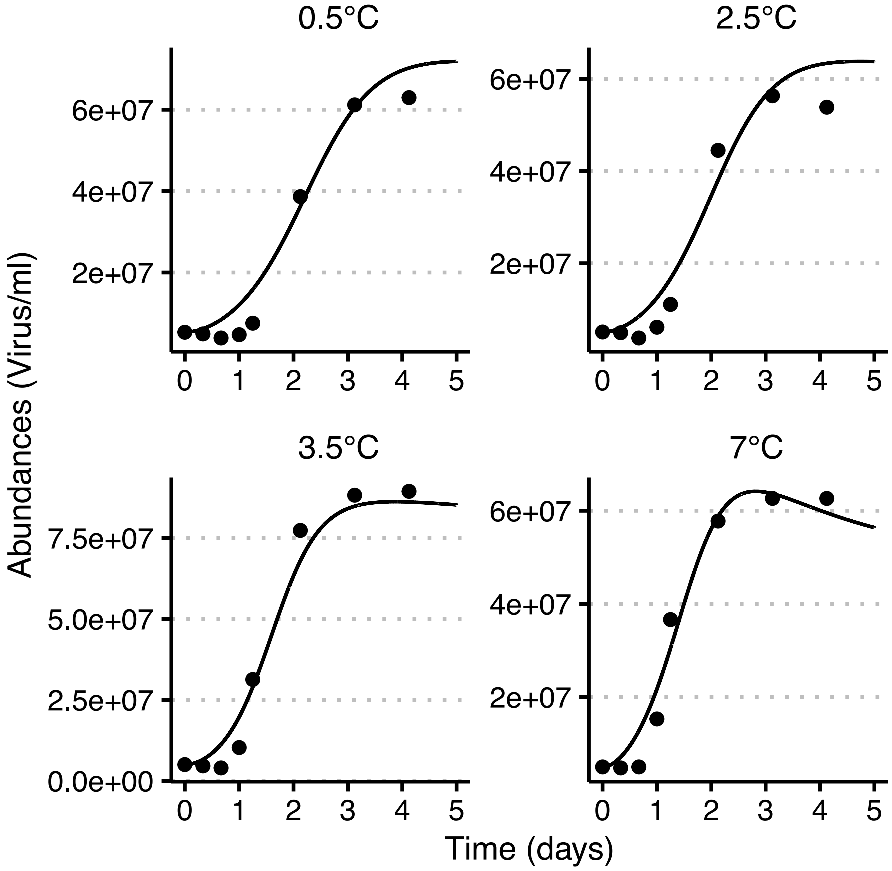


**Figure S4.** Model-data fitting for the polar virus-host pair. Virus dynamics at 0.5, 2.5, 3.5 and 7°C. Solid black lines are the model fits and black dots the data from [21].

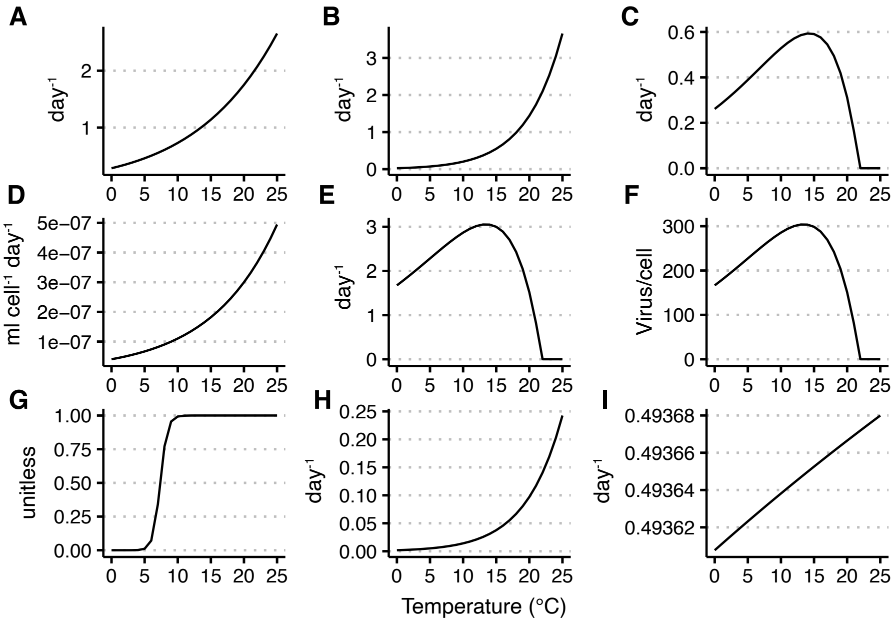


**Figure S5.** Temperature-driven hyperfunction for the host and virus life history traits estimated from the model-data fitting. A) Host gross growth rate, B) Host mortality rate, C) Host net growth rate (gross growth rate minus mortality), D) Viral adsorption rate, E) Viral lysis rate, F) Viral burst size, G) Proportion of produced non-infectious viral particle, H) Viral particle loss of infectivity and I) Viral degradation rate. More details on the hyperfunction equations can be found in [22].

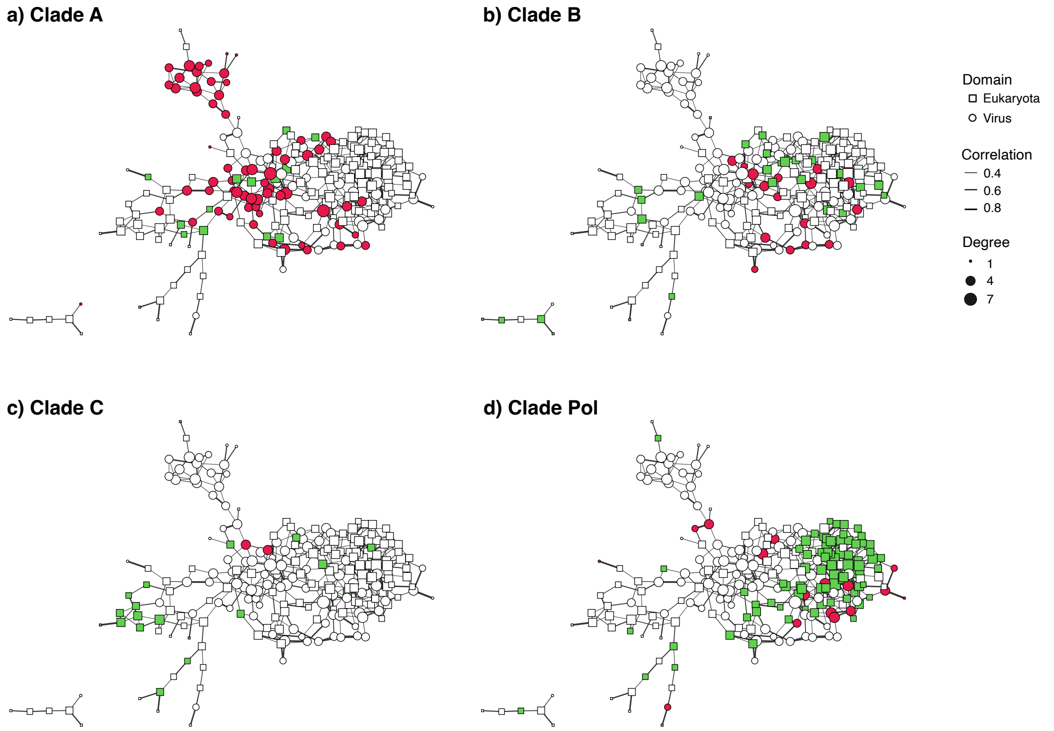


**Figure S6.** Virus-host interaction networks highlighting the **a)** clade A, **b)** clade B, **c)** clade C, and **d)** clade Pol. Networks are color-coded by viruses (red circle) and hosts (green square) for each clade. Edge width and node size indicate correlation coefficient and degree (i.e., the number of connections), respectively.

## Supplementary Tables

**Table S1.** Information about parameters used in the model from [22].

| Parameter | Signification | Units |
|---|---|---|
| $\mu$ | host division rate | day$^{-1}$ |
| $\psi$ | basal host loss rate | day$^{-1}$ |
| $K$ | carrying capacity | cell ml$^{-1}$ |
| $\phi$ | adsorption rate | ml cell$^{-1}$ day$^{-1}$ |
| $\eta$ | lysis rate | day$^{-1}$ |
| $\epsilon$ | percentage of non-infectious viruses produced | unitless |
| $\beta$ | viral burst size | virus cell$^{-1}$ |
| $\sigma$ | loss of infectivity | day$^{-1}$ |
| $\delta$ | viral decay | day$^{-1}$ |
| $\omega$ | higher-order viral losses | ml virus$^{-1}$ day$^{-1}$ |

**Table S2.** Hyper-parameter values of the temperature-driven functions for the polar host-virus pair calibrated using data from [21]. Hyper-parameter values used for the 3 other pairs can be found in [22].

| Hyper-parameter | Mic-Pol/MicV-Pol | Units |
|---|---|---|
| $A1$ | 1 $10^{11}$ | day$^{-1}$ |
| $E1$ | 7260.7 | °K |
| $A2$ | 1.99 $10^{24}$ | day$^{-1}$ |
| $E2$ | 16296 | °K |
| $K$ | 1 $10^{10}$ | cell ml$^{-1}$ |
| $\phi_K$ | 2.63 $10^{-7}$ | ml (cell day)$^{-1}$ |
| $T_\phi$ | 291.83 | °K |
| $\phi_r$ | 0.1 | °K$^{-1}$ |
| $s1$ | 2.09 $10^{9}$ | day$^{-1}$ |
| $d1$ | 5705.3 | °K |
| $s2$ | 3.05 $10^{24}$ | day$^{-1}$ |
| $d2$ | 16007 | °K |
| $T_\epsilon$ | 280.5 | °K |
| $\epsilon_r$ | 9.2464 | unitless |
| $\sigma_1$ | 5.02 $10^{22}$ | day$^{-1}$ |

**Table S3.** Scores and metrics for the 3 first axes of the PCoA of Figure 1c.

| Axis | Eig | Rel_eig | Rel_corr_eig | Brok_stick | Cum_corr_eig | Cum_br_stick |
|---|---|---|---|---|---|---|
| 1 | 13.125 | 0.2577 | 0.1564 | 0.0436 | 0.1564 | 0.0436 |
| 2 | 8.5968 | 0.1688 | 0.1036 | 0.0355 | 0.2600 | 0.0791 |
| 3 | 3.7054 | 0.0728 | 0.0465 | 0.0315 | 0.3065 | 0.1105 |
